# Pseudo–Left Ventricular Summit Premature Ventricular Contraction in a Patient With Abnormal Intraventricular Conduction

**DOI:** 10.1016/j.jaccas.2025.106530

**Published:** 2026-01-14

**Authors:** Hung Pham Nhu, Huy Nguyen The Nam, Tuan Nguyen Xuan, Dan Nguyen Van, Tung Pham Van

**Affiliations:** Hanoi Heart Hospital, Hanoi, Vietnam

**Keywords:** ablation, intraventricular conduction delay, pseudo-LV summit PVC, right ventricular outflow tract

## Abstract

**Background:**

Catheter ablation of premature ventricular complexes (PVCs) remains challenging in select scenarios, particularly those arising from the left ventricular (LV) summit or from epicardial sites.

**Case Summary:**

We report a case of PVCs with a surface electrocardiographic pattern suggestive of LV summit origin but ultimately mapped and successfully ablated in the right ventricular outflow tract (pseudo–LV summit PVC) due to the presence of subtle conduction abnormalities.

**Discussion:**

Intraventricular conduction abnormalities may have modified ventricular activation patterns, resulting in a pseudo–LV summit morphology. This case highlights the importance of a systematic mapping approach and cautious interpretation of surface ECG criteria, underscoring the need for comprehensive endocardial evaluation before pursuing epicardial ablation.

**Take-Home Messages:**

Intraventricular conduction abnormalities may alter PVC morphology and mislead ECG-based localization. A systematic, stepwise mapping approach—from endocardial to epicardial evaluation—remains essential for accurate diagnosis and effective ablation.

## Visual Summary

**Figure:**
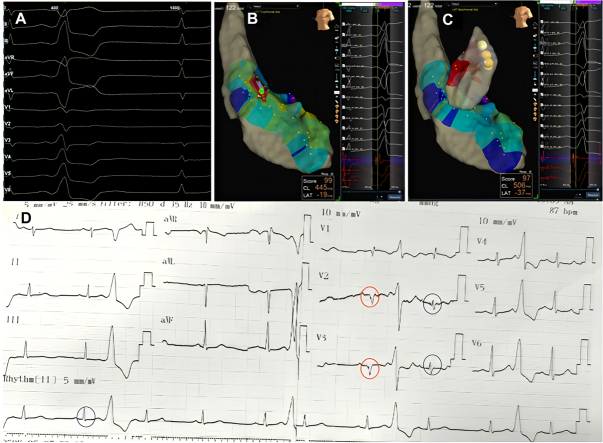

## History of Presentation

**Figure 1:**
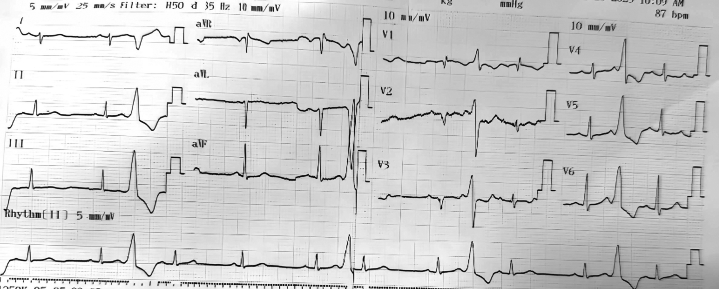
12-Lead Electrocardiogram Demonstrating Frequent Monomorphic Premature Ventricular Complexes

A 35-year-old woman presented with frequent episodes of palpitations. A 12-lead electrocardiogram was obtained (Figure 1). Twenty-four–hour Holter monitoring demonstrated a premature ventricular complex (PVC) burden of 30%. Treatment with a beta-blocker was initiated but failed to provide symptomatic or arrhythmic improvement. The patient was subsequently referred for catheter ablation.

## Past Medical History

The patient had undergone surgical closure of an atrial septal defect 10 years earlier. She remained clinically stable, with no symptoms or signs of heart failure.

## Investigations

**Figure 2:**
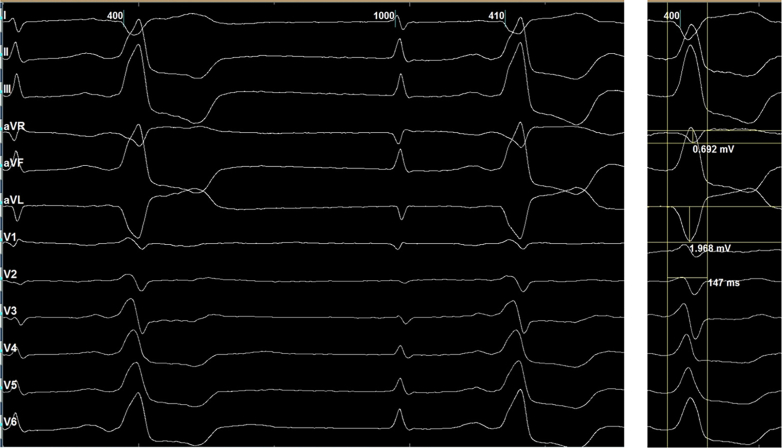
Electrocardiogram During Electroanatomic Mapping, Showing an Early Transition in Lead V_1_, a Pattern Break in Lead V_2_, and an aVL/aVR Q-Wave Ratio of Approximately 2.8

The 12-lead electrocardiogram demonstrated PVCs with a transition in lead V_1_ and a pattern break in lead V_2_, suggestive of a left ventricular (LV) summit origin.^1^ The aVL/aVR ratio was 2.8, further indicating that an epicardial approach might be required for ablation^2^ (Figure 2).

## Management

**Figure 3:**
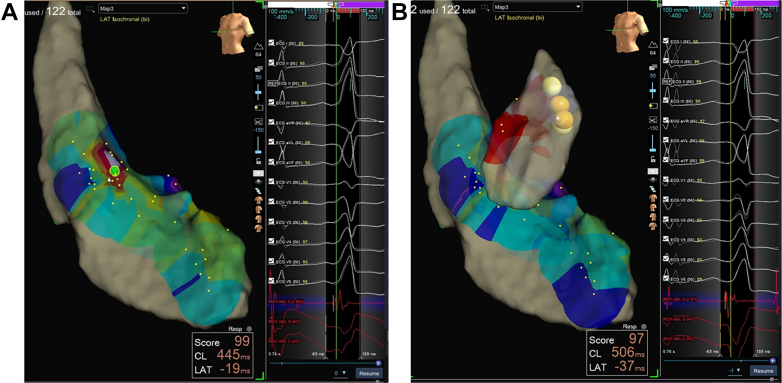
Electroanatomic Mapping of the Left and Right Ventricles (A) Three-dimensional left ventricular mapping showing the earliest activation site (green tag, 19 ms). Ablation at this site failed to eliminate the premature ventricular complexes (PVCs). (B) The earliest activation site was subsequently identified in the right ventricular outflow tract, where ablation successfully eliminated the PVCs.

**Figure 4:**
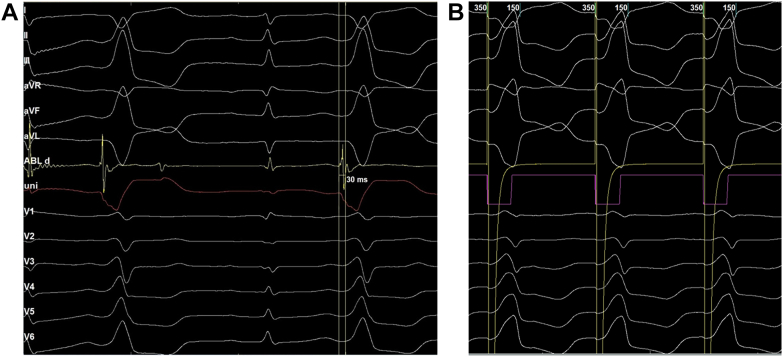
Electrogram Recordings and Pace Mapping at the Target Site (A) Earliest activation site identified in the right ventricular outflow tract, 30 ms pre-QRS. (B) Pace mapping at this site reproduced a morphology closely matching the clinical premature ventricular complexes, with a pacing score of 95%.

Endocardial mapping of the left ventricle was performed first. Neither mapping in the aortic sinuses of Valsalva nor beneath the sinuses revealed an early activation site or resulted in effective ablation (Figure 3A). The approach was then shifted to the right ventricular outflow tract (RVOT), where an earliest activation site was identified 30 ms pre-QRS, located just below the pulmonary valve (Figures 3B and 4A). Pace mapping at this site produced a morphology closely matching the clinical PVCs, with a pacing score of 95% (Figure 4B). Radiofrequency ablation was delivered at 35 W, resulting in PVC termination within approximately 5 seconds. The patient was monitored for 30 minutes post ablation with no recurrence observed and was discharged the following day.

## Discussion

The surface electrocardiogram morphology (Figures 1 and 2) did not initially suggest a RVOT origin and could have been consistent with an LV summit focus, given the early transition in lead V_1_ and the presence of a pattern break in lead V_2_. The V_2_ pattern break sign alone, however, cannot confirm an epicardial origin of PVCs, and to date, no published data have reported its sensitivity or specificity. Previous studies have described the characteristic ECG features of LV summit PVCs as including a V_2_ pattern break, a negative QRS complex in lead I, and a more negative aVL than aVR.^3^ In our case, all 3 of these features were present. The MDI (maximum deflection index) is also an indicator suggestive of an epicardial origin of PVCs. In our case, the MDI was <0.5. It should be noted, however, that an MDI ≥0.55 generally suggests a possible epicardial origin of PVCs in general, and not specifically those arising from the LV summit. Many LV summit PVCs do not exhibit an MDI >0.55. The reported diagnostic performance of MDI varies among studies. In the study by Ermengol Vallès et al, the sensitivity and specificity of MDI for identifying epicardial PVCs were 33% and 75%, respectively,^4^ indicating that MDI has high specificity but low sensitivity.

**Figure 5:**
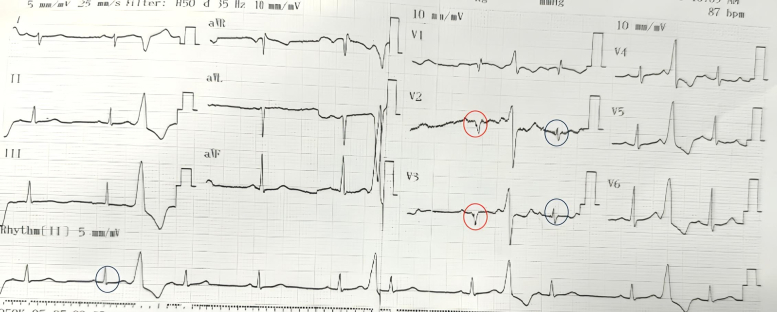
Detailed Analysis of the Baseline ECG Blue circles highlight narrow QRS complexes, most apparent in leads V_2_, V_3_, and lead II. Red circles indicate wider QRS complexes with inversion in V_2_ and V_3_, consistent with intraventricular conduction delay.

Although the ECG morphology exhibited characteristics suggestive of an LV summit origin, the earliest activation was localized to the RVOT. Pace mapping at this site reproduced a nearly identical PVC morphology (R > S in V_1_ with a pattern break in V_2_), strongly supporting an RVOT origin. This finding is particularly interesting, as PVCs generated by RVOT pacing only rarely display such a pattern. Careful inspection of the surface ECG (Figure 5) revealed subtle intraventricular conduction abnormalities, most apparent in leads V_2_ and V_3_, where QRS complexes intermittently widened and inverted (highlighted in red), indicating conduction delay. Such conduction abnormalities can significantly alter PVC morphology, potentially mimicking an LV summit origin. We have not found published reports describing this phenomenon. From an electrophysiologic standpoint, however, if a PVC originates from the right ventricle in a patient with left bundle branch block, fascicular delay, or Purkinje network injury, the impulse would first depolarize the right ventricle and then propagate through the Purkinje system. Areas with delayed or impaired conduction could modify the activation sequence and consequently alter the PVC morphology compared with typical cases.

This case reinforces the importance of a stepwise mapping strategy—starting with the left ventricle, right ventricle, and coronary sinus, and reserving epicardial access as a last resort—even when electrocardiographic criteria strongly suggest an epicardial focus with high sensitivity and specificity. Previous studies have shown that the Q-wave ratio in leads aVL and aVR is a valuable predictor of the optimal ablation site. Ratios between 1.53 and 1.74 are most commonly associated with foci located at the junction of the great cardiac vein and anterior interventricular vein, demonstrating 85% sensitivity and 98% specificity. Ratios >1.74 generally predict endocardial ablation failure, in which case an epicardial approach should be considered.^5^ Individual variations in ventricular conduction may lead to misleading surface ECG patterns, and subtle conduction disturbances are not always apparent on the ECG. These findings highlight the importance of comprehensive endocardial mapping before proceeding to more complex or invasive ablation strategies.

## Follow-Up

A 24-hour Holter ECG performed 1 month after the procedure demonstrated no recurrence of PVCs.

## Conclusions

PVC morphology can be significantly altered in the presence of intraventricular conduction abnormalities, potentially reducing the accuracy of electrocardiographic localization criteria. This case underscores the need for careful interpretation of surface ECG criteria and comprehensive mapping before considering complex or epicardial ablation strategies.

**Visual Summary:**
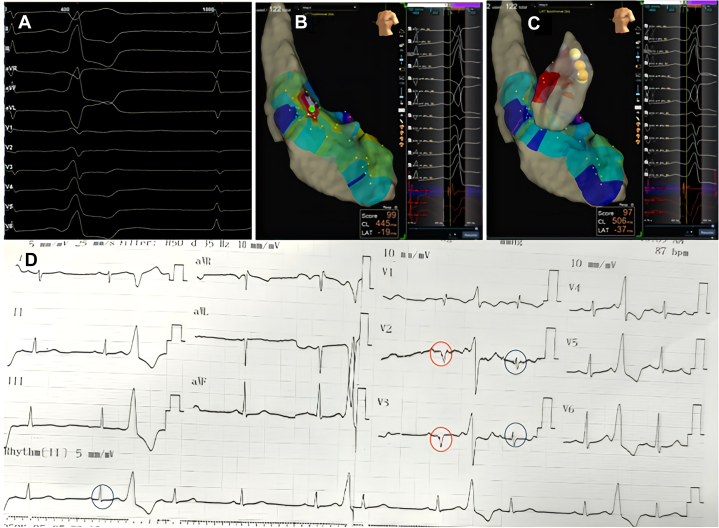
Pseudo–Left Ventricular Summit Premature Ventricular Complex (A) Surface ECG demonstrating features suggestive of a left ventricular summit origin, including a V_2_ pattern break, a negative QRS complex in lead I, and a more negative aVL compared with aVR (see also D). (B) Three-dimensional left ventricular mapping showing the earliest activation site (green tag, 19 ms), where ablation was ineffective. (C) Earliest activation subsequently identified in the right ventricular outflow tract, where ablation successfully eliminated the premature ventricular complexes. (D) Detailed analysis of the baseline ECG. Blue circles highlight narrow QRS complexes (most prominent in leads V_2_, V_3_, and II). Red circles indicate wider and inverted QRS complexes in V_2_ and V_3_, consistent with subtle intraventricular conduction delay.

## Funding Support and Author Disclosures

The authors have reported that they have no relationships relevant to the contents of this paper to disclose.

**Take-Home Messages**

• To recognize that intraventricular conduction abnormalities can alter premature ventricular complex morphology, resulting in a surface ECG appearance that may not correspond to the true site of origin.
• To emphasize a stepwise ablation strategy—beginning with endocardial mapping of the left and right ventricles, followed by coronary sinus mapping, and reserving epicardial access for refractory cases.
